# A chimeric adenovirus‐vectored vaccine based on Beta spike and Delta RBD confers a broad‐spectrum neutralization against Omicron‐included SARS‐CoV‐2 variants

**DOI:** 10.1002/mco2.539

**Published:** 2024-04-27

**Authors:** Weiqi Hong, Hong Lei, Dandan Peng, Yuhe Huang, Cai He, Jingyun Yang, Yanan Zhou, Jian Liu, Xiangyu Pan, Haiying Que, Aqu Alu, Li Chen, Jiayuan Ai, Furong Qin, Binhan Wang, Danyi Ao, Zhen Zeng, Ying Hao, Yu Zhang, Xiya Huang, Chunjun Ye, MinYang Fu, Xuemei He, Zhenfei Bi, Xuejiao Han, Min Luo, Hongbo Hu, Wei Cheng, Haohao Dong, Jian Lei, Lu Chen, Xikun Zhou, Wei Wang, Guangwen Lu, Guobo Shen, Li Yang, Jinliang Yang, Jiong Li, Zhenling Wang, Xiangrong Song, Qiangming Sun, Shuaiyao Lu, Youchun Wang, Ping Cheng, Xiawei Wei

**Affiliations:** ^1^ Laboratory of Aging Research and Cancer Drug Target State Key Laboratory of Biotherapy and Cancer Center National Clinical Research Center for Geriatrics West China Hospital Sichuan University Chengdu Sichuan China; ^2^ National Kunming High‐level Biosafety Primate Research Center Institute of Medical Biology Chinese Academy of Medical Sciences and Peking Union Medical College Kunming Yunnan China

**Keywords:** adenovirus, broad‐spectrum neutralization, intranasal vaccine, SARS‐CoV‐2

## Abstract

Urgent research into innovative severe acute respiratory coronavirus‐2 (SARS‐CoV‐2) vaccines that may successfully prevent various emerging emerged variants, particularly the Omicron variant and its subvariants, is necessary. Here, we designed a chimeric adenovirus‐vectored vaccine named Ad5‐Beta/Delta. This vaccine was created by incorporating the receptor‐binding domain from the Delta variant, which has the L452R and T478K mutations, into the complete spike protein of the Beta variant. Both intramuscular (IM) and intranasal (IN) vaccination with Ad5‐Beta/Deta vaccine induced robust broad‐spectrum neutralization against Omicron BA.5‐included variants. IN immunization with Ad5‐Beta/Delta vaccine exhibited superior mucosal immunity, manifested by higher secretory IgA antibodies and more tissue‐resident memory T cells (T_RM_) in respiratory tract. The combination of IM and IN delivery of the Ad5‐Beta/Delta vaccine was capable of synergically eliciting stronger systemic and mucosal immune responses. Furthermore, the Ad5‐Beta/Delta vaccination demonstrated more effective boosting implications after two dosages of mRNA or subunit recombinant protein vaccine, indicating its capacity for utilization as a booster shot in the heterologous vaccination. These outcomes quantified Ad5‐Beta/Delta vaccine as a favorable vaccine can provide protective immunity versus SARS‐CoV‐2 pre‐Omicron variants of concern and BA.5‐included Omicron subvariants.

## Graphical abstract

A chimeric adenovirus‐vectored vaccine named Ad5‐Beta/Delta was developed by introducing the RBD derived from the Delta variant (containing L452R and T478K) into the full‐length spike protein of Beta variant. Immunization with Ad5‐Beta/Delta induces broad‐spectrum neutralizing activities against BA.5‐included SARS‐CoV‐2 subvariants. Combination of intramuscular and intranasal delivery of Ad5‐Beta/Delta induces superior systemic and mucosal immune responses. In addition, Ad5‐Beta/Delta as a booster shot is able to elicit stronger protective immunity in heterologous vaccination after two injections of mRNA‐ and subunit protein‐based vaccines.

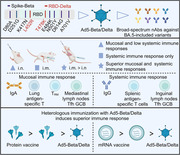

## 1 INTRODUCTION

New strains of severe acute respiratory coronavirus‐2 (SARS‐CoV‐2) are constantly emerging, imposing significant strains on global healthcare infrastructures. Successive waves of widespread transmission with remarkable velocity have been initiated by earlier reported variants of concern (VOCs), among them Alpha (B.1.1.7), Beta (B.1.351), Gamma (P.1), and Delta (B.1.617.2). Most notably, the Omicron (B.1.1.529) variant and its associated sublineages, including BA.1, BA.2 (BA.2.12.1), BA.3, BA.4/5, BQ.1.1, as well as the XBB‐lineage variants (e.g., XBB.1.5, XBB.1.16, XBB.2.3), EG.5.1, and HV.1, exhibit an extensive array of mutations within the spike protein (SP), leading to an increased absolute count of individuals afflicted with COVID‐19.^1^, ^2^, ^3^, ^4^, ^5^ The majority of these variants have profoundly undermined the neutralizing efficacy conferred by existing COVID‐19 vaccines, which were formulated in response to the original SARS‐CoV‐2 strain.^6^, ^7^, ^8^, ^9^, ^10^, ^11^ In light of the limited durability of immunity and compromised neutralization capabilities observed in first‐generation COVID‐19 vaccines targeting SARS‐CoV‐2 variants, developing a novel range of COVID‐19 vaccines that provide comprehensive immunity toward a wide array of SARS‐CoV‐2 VOCs, with a specific emphasis on the Omicron variant and its many subvariants, is crucial.

Employing spike antigen updates tailored to specific variants could represent an alternative approach. Nonetheless, earlier investigations have revealed that vaccines matched to the Omicron BA.1 variant induce only a restricted cross‐neutralizing humoral immune response.^12^ Additionally, studies have shown that administering a booster matched to Omicron BA.1 does not confer any notable advantages in eliciting higher levels of neutralizing antibodies (Abs) when contrasted to boosters employing existing messenger RNA (mRNA)‐1273 vaccines.^13^ Subvariants of the SARS‐CoV‐2 Omicron, BA.2.12.1, BA.4, and BA.5, have shown the capacity to avoid the immunological response triggered by prior BA.1 infection. This highlights the restriction of BA.1‐matched vaccination boosters to offering comprehensive protection against developing Omicron subvariants.^1^ Furthermore, such a strategy entails significant time and resource investments, resulting in inefficiencies and wastage of valuable resources. Therefore, the objective is to create a next‐generation universal COVID‐19 vaccination that can effectively neutralize various variations, including those encompassed by Omicron and may offer a prudent approach to address the breakthrough infections stemming from compromised immunity to SARS‐CoV‐2 variants. The SP of the Beta variant, which has alterations like K471N, E484K, and N501Y, is a strong possibility for stimulating strong Ab responses with cross‐neutralizing potential against a diverse array of pre‐Omicron VOCs. Several human studies have been registered to assess the safety and efficacy of antigens derived from Beta variants.^14^, ^15^, ^16^, ^17^ Nevertheless, it is noteworthy that the Omicron and its subvariants have significantly diminished the neutralizing efficacy conferred by the Beta variant. In response to this challenge, we have successfully developed a trimeric protein vaccine derived from the receptor‐binding domain (RBD) of the Delta sequence. This vaccine has demonstrated the capacity to stimulate elevated levels of broad‐spectrum neutralizing Abs (nAbs). Importantly, our studies have revealed that mutations such as L452R and T478K in the Delta‐derived vaccine contribute positively to the Ab response elicited against the Omicron variant.^18^ Hence, we hypothesized that combining the spike sequence from the Beta variant with RBD from Delta variants to create a bivalent antigen that possesses the capacity to effectively neutralize an extensive variety of SARS‐CoV‐2 variants, including Omicron.

In addition to antigen selection, another crucial aspect of enhancing the efficacy of respiratory virus vaccines involves optimizing vaccine delivery to stimulate mucosal and systemic immunity. Currently, most of the COVID‐19 vaccinations authorized for emergency use globally are given by intramuscular (IM) delivery, which has proven effective in safeguarding against symptomatic infection, severe disease, and mortality.^19^, ^20^, ^21^, ^22^, ^23^ Nevertheless, the administration of COVID‐19 vaccinations by IM injection may not completely eliminate the shedding and transmission of the SARS‐CoV‐2, as it fails to elicit mucosal immunity necessary for safeguarding against upper‐airway infections.^24^, ^25^ Previous researches have indicated that intranasal (IN) immunization with adenoviral‐vector vaccines can establish local mucosal‐tissue‐resident immunity in proximity to the site of SARS‐CoV‐2 entry.^26^, ^27^, ^28^, ^29^, ^30^, ^31^ Secretory polymeric IgA, a predominant component of mucosal immunity, has been demonstrated to neutralize invading SARS‐CoV‐2 at the infection site.^29^, ^32^, ^33^ Furthermore, lung tissue‐resident memory T (T_RM_) cells, localized directly at barrier tissues, serve as the frontline defense against SARS‐CoV‐2. These cells possess the capability to promptly exert both innate and adaptive functions.^26^, ^32^, ^34^ Nonetheless, single IN delivery has been observed to elicit relatively limited cellular immune responses in systemic immunity.^26^, ^35^ The research intended to investigate whether combining IM and IN administration may generate strong mucosal and systemic immunity to protect toward SARS‐CoV‐2 variants encompassing those from the Omicron lineage.

Herein, we created a next‐generation COVID‐19 chimeric vaccine, called Ad5‐Beta/Delta vaccine by combining RBD of the Delta variant with the complete spike antigen of the Beta variant. Our research shows that administering the Ad5‐Beta/Delta vaccine via IM and IN routes results in long‐lasting humoral immune responses. These responses are marked by elevated levels of neutralizing Abs that may effectively neutralize a wide range of Omicron‐included variants, including BA.5. Moreover, the combination of IM and IN delivery of the Ad5‐Beta/Delta vaccine induces superior mucosal and systemic immunity. In conclusion, our study demonstrates that the Ad5‐Beta/Delta vaccine displays a favorable boosting effect when administered as an additional booster shot in heterologous vaccination regimens. Theses underscore the potential of our chimeric vaccine as a viable choice for preventing SARS‐CoV‐2 mutations.

## 2 RESULTS

### 2.1 Construction and characterization of chimeric Ad5‐Beta/Delta vaccine

**FIGURE 1:**
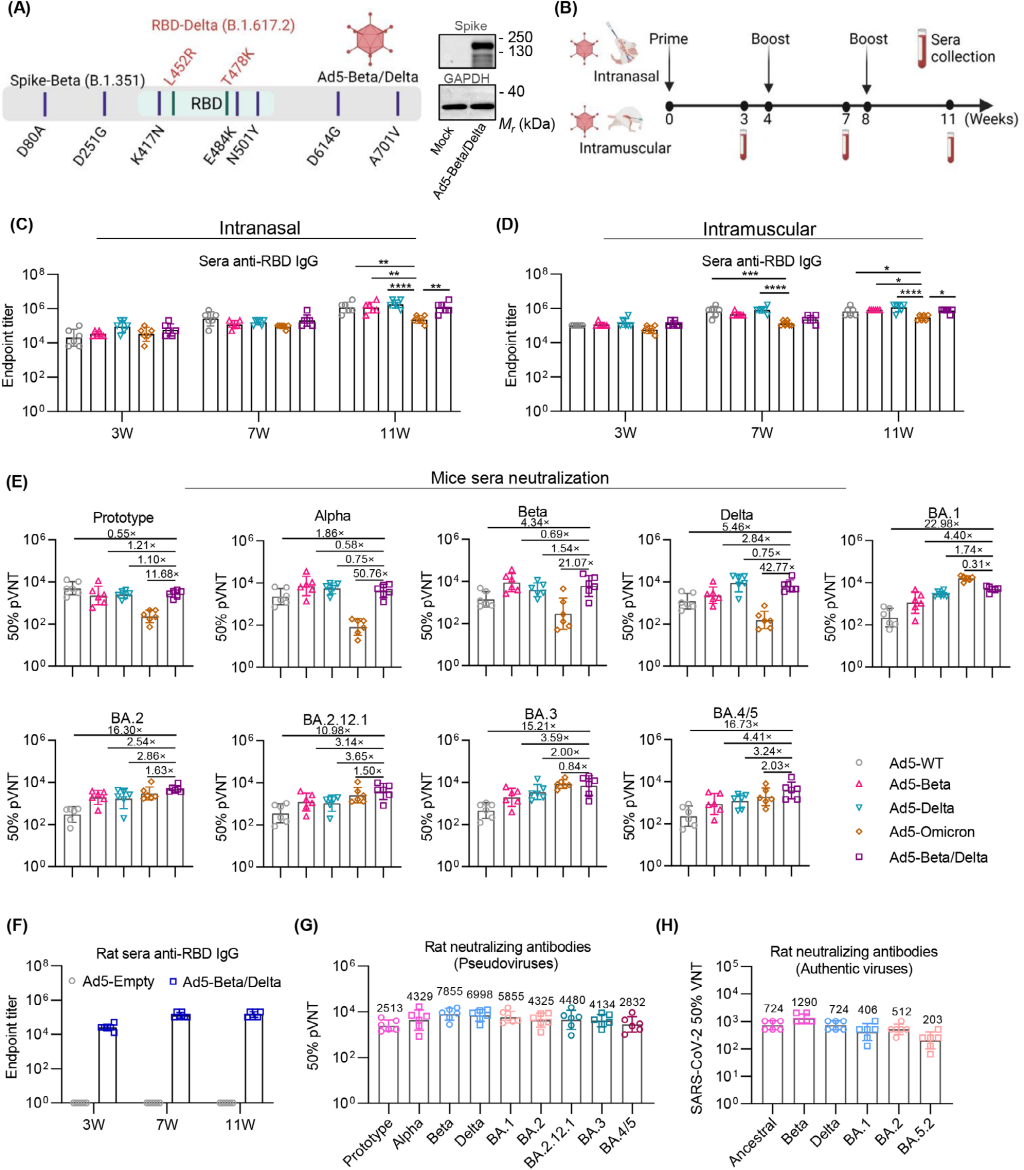
Ad5‐Beta/Delta vaccine induces broad‐spectrum neutralization against BA.5 Omicron‐included SARS‐CoV‐2 variants. (A) The illustrative representation of the design of Ad5‐Beta/Delta vaccine (left). The RBD derived from Delta variant (encompassing L452R and T478K mutations) was introduced into the entire SP from Beta variants. WB of expression of SP in HEK‐293T cells in the absence or presence of Ad5‐Beta/Delta (right). (B) The diagram illustrating the process for immunization and collection of sera. (C and D) Endpoint titers of anti‐RBD IgG in mouse sera elicited by IN (C) or IM (D) immunization with adenovirus‐vectored vaccines (*n* = 6 mice per group). (E) Neutralizing Ab titers in serum samples from mice immunized with various adenovirus‐vectored vaccines by IN delivery (*n* = 6 mice per group). (F) Endpoint titers of anti‐RBD IgG in sera from rats IN immunized with 2 × 10^10^ VPs of Ad5‐Empty or Ad5‐Beta/Delta vaccine (*n* = 6 rats per group). (G and H) Titers of neutralizing Abs in the sera of rats (*n* = 6 rats per group) immunized with Ad5‐Beta/Delta vaccine against SARS‐CoV‐2 pseudoviruses (G) and authentic viruses (H). The serum samples in (E) and (G) were gathered 11 weeks subsequent to the initial immunization. Data are presented as geometric mean values ± SD. *p* Values were calculated employing two‐way ANOVA analysis followed by Tukey's multiple comparisons test in (C and D). *p < 0.05; **p < 0.01; ***p < 0.001; ****p < 0.0001; ns, not significant.

Based on the previous report that good immunogenicity of spike of Beta variant and RBD of Delta variant, we developed a chimeric adenovirus vector vaccine by introducing amino acid mutations of RBD of Delta variant into the entire SP of Beta variant (Figure 1A, left). In the subsequent material, we referred to this chimeric vaccine as Ad5‐Beta/Delta. The intact expression of SP was verified by western blot with a polyclone anti‐spike Ab after 293T cells were exposed to the Ad5‐Beta/Delta (Figure 1A, right). To compare the immunogenicity of spikes from other variants, monovalent adenovirus vector vaccines were created to encode the SP of ancestral (Ad5‐WT), Beta (Ad5‐Beta), Delta (Ad5‐Delta), and Omicron BA.1 (Ad5‐Omicron) strains. The expression of each SP was identified using enzyme‐linked immunosorbent assay (ELISA) (Figure S1). The corresponding encoded SP can be expressed after being infected with various adenovirus vectors, and there is no significant difference in expression level, which excludes the possibility of the different immunogenicity of each spike caused by inconsistent protein expression.

### 2.2 Ad5‐Beta/Delta vaccine elicits broad‐spectrum neutralizing capacities against BA.5 Omicron‐included SARS‐CoV‐2 variants

To ascertain the immunogenicity of the Ad5‐Beta/Delta vaccine, a prime‐boost regimen consisting of 5 × 10^9^ virus particles (VPs) of the Ad5‐Beta/Delta vaccine administered IN or IM to 6‐week‐old BALB/c mice was implemented. The mice were immunized every 28 days apart with Ad5‐Empty serving as a control (Figure 1B). To compare the immunogenicity of spike from other variants, monovalent Ad5 vector vaccines encoding the SP of ancestral, Beta, Delta, and Omicron (BA.1) strains were injected with same prime‐boost immunized schedule. Serum specimens were obtained at 3, 7, and 11 weeks after the first immunization to analyze the kinetics of the RBD‐specific binding Ab response. Both IN and IM delivery of adenovirus vector vaccines can generate great levels of RBD‐specific IgG following one single dose (Figures 1C and D). Although the Omicron spike showed lower antigenicity, immunization with all of adenovirus‐vectored vaccines via IN or IM delivery could induce sustained binding Abs (Figures 1C and D).

The subsequent step, the pseudovirus neutralization assay was carried out to evaluate the nAbs in sera from mice IN that had received adenovirus‐vectored vaccines. Each VOC vaccination induced strong neutralizing activity unique to the corresponding VOCs (Figure 1E). In line with previous studies, neutralization capacity induced by the adenovirus vector vaccine designed based on the original spike (Ad5‐WT) was significantly impaired by variants, especially by Delta variant and BA.5‐included Omicron subvariants, and adenovirus vector expressing spike of Omicron BA.1 variant (Ad5‐Omicron) induced a limited‐cross neutralizing activities against all pre‐Omicron VOCs. Compared with other adenovirus vectors, immunization of Ad5‐Beta/Delta vaccine elicited high broad‐spectrum nAbs in mice. These Abs were able to effectively prevent the infection of all pseudoviruses in the study (Figure 1E). The group that received Ad5‐Beta/Delta vaccination had geometric mean titers (GMTs) of 50% neutralization against several pseudoviruses as follows: prototype (2729), Alpha (4171), Beta (6130), Delta (6572), Omicron (BA.1) (5031), BA.2 (4876), BA.2.12.1 (3836), BA.3 (6921), and BA.4/5 (3484) (Figure 1E). Of note, Ad5‐Beta/Delta vaccine induced the greatest levels of neutralizing Abs against BA.4/5 subvariant compared with other adenovirus vector vaccines (Figure 1E). An antigenic map from pseudovirus neutralization data (Figure 1E) was generated by multidimensional scaling algorithm^36^, ^37^ (Figure S2). Consistent with previous studies,^36^, ^38^ pre‐Omicron variants grouped closely in one cluster, and Omicron subvariants formed distinct antigenic outliers in the map. Similar to the results of pseudovirus neutralization assay, homologous sera showed high titers of neutralization against corresponding variants, manifested by the nearest distance between sera neutralizing Abs and the variant corresponding to the antigen. The sera obtained from mice vaccinated with Ad5‐Beta/Delta vaccine located halfway between the clusters of pre‐Omicron and Omicron subvariants on the antigenic map, suggesting its ability to induce broad‐spectrum neutralization against pre‐Omicron and BA.5‐included Omicron subvariants.

In addition, IN immunization with the Ad5‐Beta/Delta vaccine showed good immunogenicity and elicited high levels of sera neutralizing Abs against several variants in rats (Figures 1F–H). In the pseudovirus neutralization assay, the fifty percent neutralization GMTs to the prototype, Alpha, Beta, Delta, BA.1, BA.2, BA.2.12.1, BA.3, BA.4/5 variants reached 2513, 4329, 7855, 6998, 5855, 4325, 4480, 4134, and 2832, respectively (Figure 1G). The authentic neutralization experiments were performed to further ascertain the neutralizing activities induced by Ad5‐Beta/Delta, and the GMTs of 50% neutralization to live ancestral, Beta, Delta, Omicron BA.1, BA.2, and BA.5.2 viruses were determined to 724, 1290, 724, 406, 512, and 203, respectively, highlighting the strong broad‐spectrum neutralization potency (Figure 1H).

### 2.3 IN delivery of Ad5‐Beta/Delta vaccine induces superior airway immune response

**FIGURE 2:**
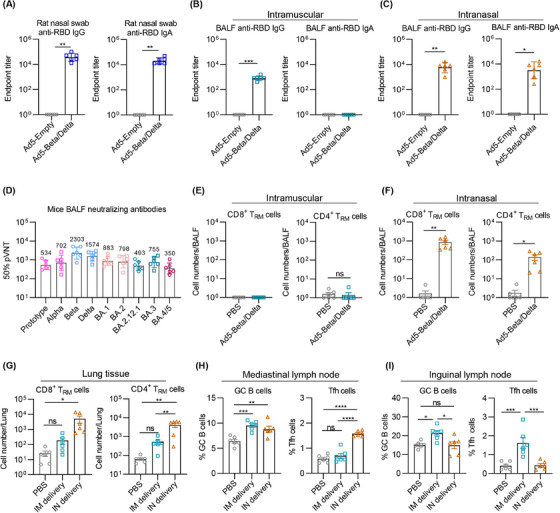
IN immunization with Ad5‐Beta/Delta vaccine elicits stronger mucosal immunity. (A) Endpoint titers of IgG (left) and IgA (right) in rat nasal swab samples that collected at 11 weeks after the first vaccination (*n* = 6 rats each group). (B and C) Endpoint titers of anti‐RBD IgG and IgA Ab in BALF from mice IM (B) or IN (C) vaccinated with Ad5‐Beta/Delta vaccine at 11 weeks postimmunization (*n* = 6 mice each group). (D) Titers of neutralizing Abs targeting pseudoviruses in BALF specimens from IN immunization group (*n* = 6). (E–G) The absolute number of CD8^+^ and CD4^+^ T_RM_ cells in BALF (E and F) and lung tissues (G) from vaccinated mice. T_RM_ cells were gated on CD44^+^CD69^+^CD103^+^ CD4^+^ or CD8^+^ (*n* = 6 mice each group). (H‐I) The prevalence of Tfh (CD4^+^CXCR5^+^PD‐1^+^) and GC B cells (CD19^+^GL7^+^CD95^+^) in the mediastinal lymph nodes (H) and inguinal lymph nodes (I) (*n* = 6 mice per group). The data are manifested in sets (A–D) as geometric mean values ± SD, and in sets (E–I) as means with SEM. Unpaired Student's *t*‐tests were deployed to ascertain *p* values in (A–C and E–F), while one‐way ANOVA with Tukey's multiple comparisons test was employed in (G–I). *p < 0.05; **p < 0.01; ***p < 0.001; ****p < 0.0001; ns, not significant.

We further evaluated the immune response caused through the Ad5‐Beta/Delta vaccine in the respiratory mucosa. The rats IN immunized with Ad5‐Beta/Delta vaccine showed robust binding Ab responses in nasal swab samples, manifested by high levels of IgG and IgA (Figure 2A). For mice, bronchoalveolar lavage fluids (BALF) were collected at 11 weeks to detect the titers of RBD‐specific IgG and IgA. Specific IgG Abs could be elicited by both IN and IM immunization, while the titers in the IM group were declined contrasted to IN group (Figure 2B, left and 2C, left). Of note, RBD‐specific IgA could only reliably be detected in the trachea‐lung washes from the mice in IN group (Figure 2B, right and 2C, right). IN immunization with Ad5‐Beta/Delta manifested potent broad‐spectrum neutralizing abilities against various SARS‐CoV‐2 variants in the respiratory tract. GMTs for 50% neutralization against various pseudoviruses, including prototype, Alpha, Beta, Delta, BA.1, BA.2, BA.2.12.1, BA.3, and BA.4/5 pseudoviruses reached 534, 702, 2303, 1574, 883, 798, 493, 755, and 350, respectively (Figure 2D).

Besides IgA Ab, T_RM_ cell response is another dominant composition of mucosal immunity in host defense, such that it could respond immediately to prevent pathogen infection. Thus, we next investigate whether our Ad5‐Beta/Delta vaccine could induce the respiratory mucosal T_RM_ cells. The cells in BALF were collected to analyze of the number of T_RM_. Both CD8^+^ and CD4^+^ T_RM_ cells (CD44^+^CD103^+^CD69^+^) were increased after the IN delivery of Ad5‐Beta/Delta vaccine, whereas the IM immunization could not induce significant T_RM_ response in airway (Figures 2E and F). In lung tissues, although the amounts of T_RM_ cells were slightly increased after IM immunization with the Ad5‐Beta/Delta vaccine, there were no statistical variations between groups of PBS and IM Ad5‐Beta/Delta (Figure 2G). Compared with IM delivery, IN immunization remarkably enhanced the recruitment of CD8^+^ and CD4^+^ T_RM_ cells (Figure 2G). The findings showed that IN vaccination, as opposed to IM immunization, may stimulate a versatile immune response in the respiratory mucosa. We also examined the germinal center (GC) and T follicular helper (Tfh) cell response, which are essential for long‐term protective immunity. IM and IN administration enhanced the proportion of GC B (CD19^+^GL7^+^CD95^+^) cells, but only IN immunization increased the number of Tfh (CD4^+^CXCR5^+^PD‐1^+^) cells in mediastinal lymph nodes (Figure 2H). In contrast, IM, but not the IN immunization with Ad5‐Beta/Deta vaccine remarkably increased the amount of GC B cells and Tfh cells in inguinal lymph nodes (Figure 2I), suggesting the combination of IM and IN delivery might have induced stronger systemic and respiratory immunity.

### 2.4 Combination of IM and IN immunization of Ad5‐Beta/Delta vaccine elicits superior mucosal and systemic immune responses

**FIGURE 3:**
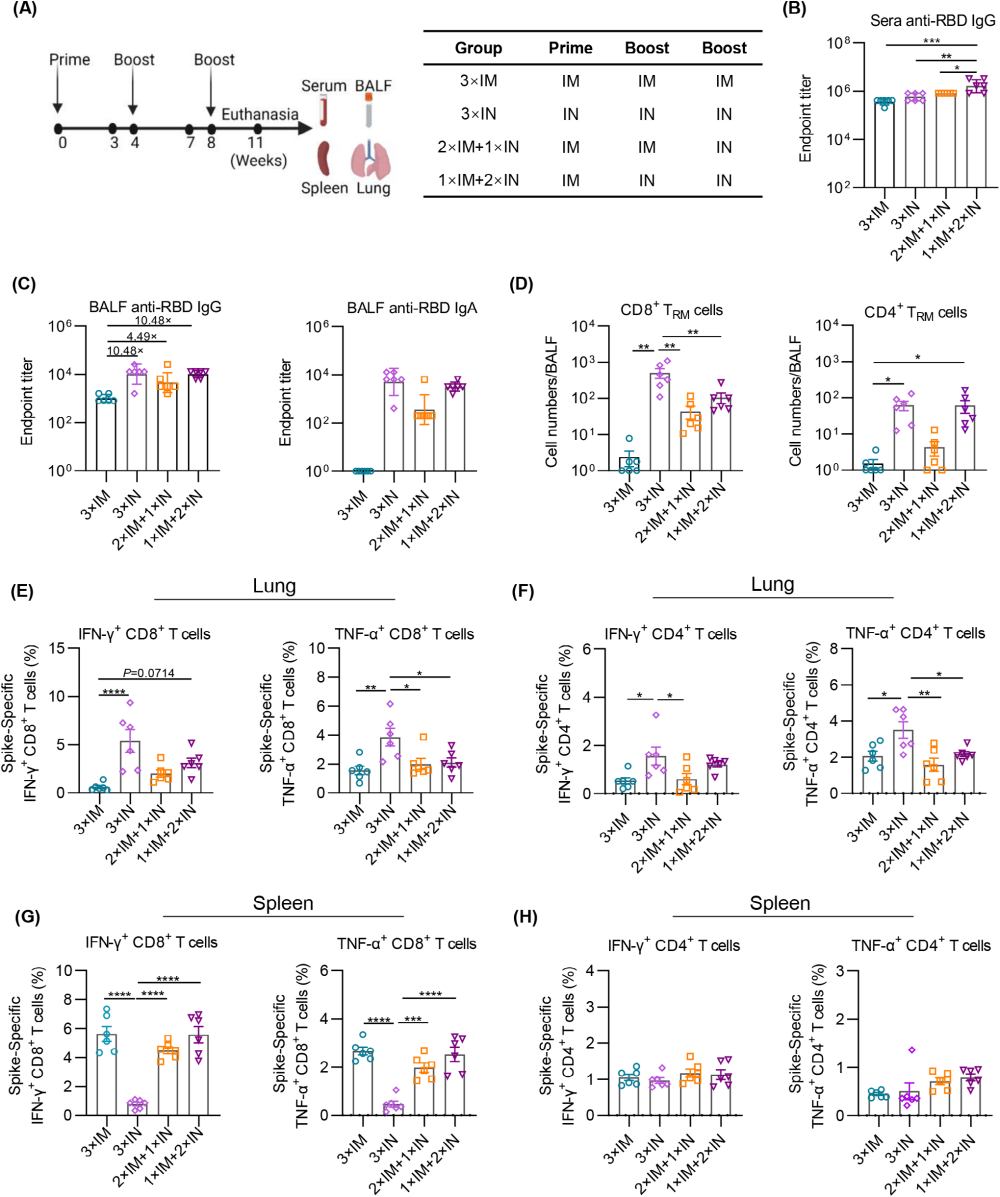
Combination of IM and IN immunization elicits superior mucosal and systemic immune response. (A) The diagram illustrating the vaccination and sample collecting protocol. (B and C) Endpoint titers of anti‐RBD IgG in sera (B), IgG and IgA in BALF (C) from mice immunized with different programs. (D) The prevalence of CD8^+^ (left) and CD4^+^ (right) T_RM_ cells in BALF from vaccinated mice. (E–H) The percentage of spike specific IFN‐γ or TNF‐α‐producing memory T cells in lung (E and F) and in spleen (G and H) were analyzed after stimulation with peptide pools for SARS‐CoV‐2 spike. Samples in (B–H) were all collected at 11 weeks after the first vaccination. *n* = 6 mice each group in (B–H). The data are displayed in the form of geometric mean values ± SD in (B and C) and mean values ± SEM in (D–H). The analysis of *p* values was performed employing one‐way ANOVA, and then Tukey's multiple comparison post hoc test was applied. *p < 0.05; **p < 0.01; ***p < 0.001; ****p < 0.0001; ns, not significant.

To investigate whether the integration of IM and IN immunization could induce a superior immune response, we then immunized mice with different immunization programs (Figure 3A). BALB/c mice received an IM injection of the Ad5‐Beta/Delta vaccination on days 0 and 28, and then received three‐does IN immunization on day 56 (2×IM+1×IN). In addition, mice were also IM immunized and followed by two dose boosts via IN delivery (1×IM+2×IN). The mice vaccinated with three doses of Ad‐Beta/Delta vaccine via IM (3×IM) or IN (3×IN) delivery were used as control. The immunization of 1×IM+2×IN elicited the greatest levels of sera RBD‐specific IgG Ab between all groups (Figure 3B). All types of immunization programs can induce great levels of RBD‐specific IgG in BALF, while three IN immunizations (3×IN) and one IM followed by two IN immunizations (1×IM+2×IN) elicited more IgG in the airway (Figure 3C, left). As expected, IgA in BALF could be reliably detected in all groups with one or more IN immunizations, and higher levels of IgA were elicited by 3×IN and 1×IM+2×IN immunization (Figure 3C). Consistent with the results of IgA levels, the counts of CD8^+^ and CD4^+^ T_RM_ cells were significantly increased in 3×IN and 1×IM+2×IN group (Figure 3D), indicating that superior mucosal immune response could be induced by at least twice IN immunization.

Next, we analyzed the antigen‐specific T cells in the lung tissue using intracellular cytokine staining (ICS). The lung tissues were isolated for preparation of single‐cell suspension and ex vivo with a full‐length spike peptide pool to determine the generation of intracellular IFN‐γ and TNF‐α. In line with previous papers,^26^, ^27^, ^28^, ^29^, ^30^, ^31^ at least one dose of IN immunization can induce a significantly elevated number of antigen‐specific T cells in the respiratory tract, whereas IM immunization hardly elicit a robust antigen‐specific T cell immune response in lung tissues (Figures 3E and F). To more comprehensively evaluate the Ad5‐Beta/Delta vaccine‐induced T cellular response, we ascertained systemic antigen‐specific T cells in spleen tissues. 3×IM but not 3×IN immunization induced a robust systemic spike‐specific CD8^+^ T cell response (Figures 3G and H). Of note, both immunizations of 2×IM+1×IN and 1×IM+2×IN overcome this shortcoming that increased the frequency of IFN‐γ and TNF‐α secreted systemic CD8^+^ T cells (Figures 3G and H). These above results demonstrated that a integration of IM and IN immunization could manifest a more stronger immune response, both at the mucosal and systemic immune levels, against infection triggered by Omicron‐included SARS‐CoV‐2 variants.

### 2.5 Ad5‐Beta/Delta vaccine as a booster shot elicits a stronger immune response in the heterologous immunization

**FIGURE 4:**
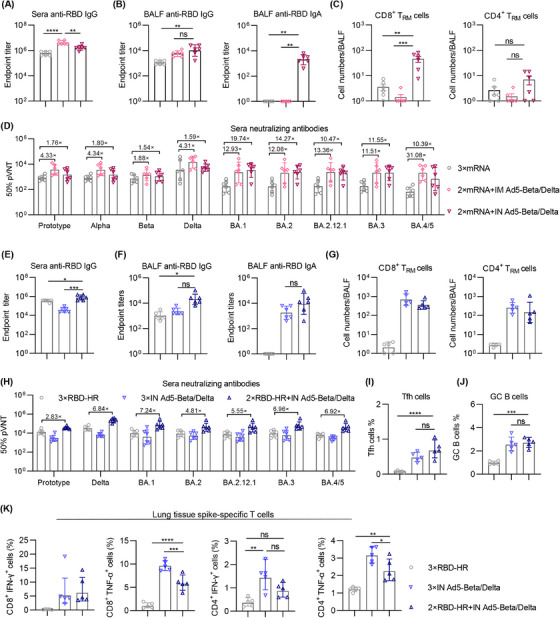
Ad5‐Beta/Delta vaccine as a booster shot in heterologous immunization enhances immune response. (A and B) Endpoint titers of anti‐RBD IgG in sera (A), IgG and IgA in BALF (B) from mice immunized with three doses of mRNA (3×mRNA), two doses of mRNA followed by IM (2×mRNA+IM Ad5‐Beta/Delta) or IN immunization (2×mRNA+IN Ad5‐Beta/Delta) of Ad5‐Beta/Delta vaccine (*n* = 6 mice per group). (C) The number of CD8^+^ and CD4^+^ T_RM_ in BALF from vaccinated mice. (D) Neutralization toward pseudoviruses in sera from mice with homologous immunized with mRNA vaccine or heterologous vaccinated with mRNA and Ad5‐Beta/Delta vaccines. (E and F) Endpoint titers of anti‐RBD IgG in sera (E), IgG and IgA in BALF (F) from mice IM injected with two doses of RBD‐HR/trimer followed by IN immunization with Ad5‐Beta/Delta vaccine (2×RBD‐HR + IN Ad5‐Beta/Delta), the mice IM immunized with three doses of RBD‐HR/trimer (3×RBD‐HR), or intranasally immunized with three doses of Ad5‐Beta/Delta (3×IN Ad5‐Beta/Delta) were used as control (*n* = 6 mice per group). (G) The number of CD8^+^ and CD4^+^ T_RM_ in BALF from vaccinated mice. (H) Neutralizing Abs in sera from mice with homologous or heterologous subunit protein vaccination. (I and J) Tfh (I) and GCB (J) cell frequencies in the mediastinal lymph nodes. (K) The proportion of memory T cells in lung tissue that produce INF‐ γ or TNF‐α in response to specific spikes. The data are manifested in the following ways: (A, B, D, E, F, and H) as geometric mean values ± SD, and (C, G, and I–K) as mean values ± SEM. The analysis of *p* values was performed employing one‐way ANOVA analysis followed by Tukey's multiple comparison post hoc test. *p < 0.05; **p < 0.01; ***p < 0.001; ****p < 0.0001; ns, not significant.

SARS‐CoV‐2 mRNA‐based vaccines have been approved for emergence and widely used around the world.^19^, ^39^ The mRNA vaccine's neutralizing powers were significantly reduced by several Omicron forms of SARS‐CoV‐2 variants, affecting its efficiency.^8^, ^9^ Furthermore, the mRNA vaccines are mostly delivered by IM injection, which reduces the prospect and applications of mucosal immunization. Therefore, we next investigated whether our Ad5‐Beta/Delta vaccine could be employed as an extra heterologous booster shot in a sequential immunization program. The BALB/c mice received an IM injection of an mRNA vaccination containing the full‐length SP on day 0 and day 28, as previously described.^18^ This is followed by a third‐dose immunization of Ad5‐Beta/Delta vaccine via IN (2×mRNA+IN Ad5‐Beta/Delta) or IM (2×mRNA+IM Ad5‐Beta/Delta) delivery on day 56. As expected, the RBD‐specific IgG titers in sera were remarkably improved by heterologous IN or IM immunization with Ad5‐Beta/Delta vaccine than homologous mRNA vaccination (Figure 4A). Moreover, heterologous IN immunization with the Ad5‐Beta/Delta vaccine induced superior airway mucosal immune responses, including higher BALF IgG and IgA titers, and more frequency of T_RM_ cells (Figures 4B and C). Additionally, heterologous immunization induced higher neutralization against SARS‐CoV‐2 pseudoviruses in the panel (Figure 4D). These outcomes indicated that our Ad5‐Beta/Delta vaccine shows potential as a strong option for heterologous immunization after vaccination with mRNA vaccine, especially via IN delivery route.

We also wonder whether the Ad5 Beta/Delta vaccine could augment the immune response produced by prior delivery of a subunit protein vaccine. The RBD‐HR/trimer is a recombinant vaccination that consists of a trimeric form of the RBD protein produced from the Delta variant. This vaccine contains L452R and T478K that we previously developed.^18^ We have previously reported that the RBD‐HR/trimer can elicit a strong humoral and cellular immune response and exhibits broad‐spectrum neutralizing ability for the Omicron variant.^18^ Nevertheless, protein vaccines are generally administered mainly IM, neglecting the protective effect of mucosal immunity on the organism. In the current study, we IN vaccinated with Ad5‐Beta/Delta vaccine in mice that have IM received two doses of RBD‐HR/trimer (2×RBD‐HR+IN Ad5‐Beta/Delta). As expected, sequential immunization enhanced robust systemic and respiratory immune responses than homologues subunit protein vaccination (Figures 4E–K). Although three doses of IN Ad5 Beta/Delta vaccinations induced considerable respiratory immunity as sequential immunization, the titers of binding and neutralizing Abs in sera were remarkably increased in heterologous immunization group (Figures 4E and H). The above results suggest that Ad5‐Beta/Delta vaccine produces good mucosal and systemic immune responses as a third dose in heterologous IN immunization, and the Ad5‐Beta/Delta vaccine could be an important candidate for third dose heterologous immunization.

## 3 DISCUSSION

The ongoing development of SARS‐CoV‐2 VOCs significantly hindered the effectiveness of existing COVID‐19 vaccinations. Despite attenuated pathogenicity than different SARS‐CoV‐2 strains,^40^ Omicron variants with many mutations contribute to a larger number of COVID‐19 cases, placing a significant strain on global healthcare systems. This critical scenario requires the creation of a new kind of universal COVID‐19 vaccination that can effectively combat the most prevalent variations, particularly the Omicron variant and its subvariants. Therefore, we engineered an adenovirus‐vector chimeric vaccine by inserting the RBD sequence obtained from Delta variant into the SP of the Beta strain, named Ad5‐Beta/Delta. Vaccination of this chimeric vaccine elicited sustained humoral immune response and strong mucosal immunity with great titers of broad‐spectrum neutralizing Abs against several pre‐Omicron VOCs and BA.5‐included Omicron subvariants. Importantly, Ad5‐Beta/Delta vaccine can provide effective protection in heterologous vaccination as an extra booster shot.

In the previous studies, we and others have reported that vaccination with mutated protein of variants exhibits better neutralizing capacity against the corresponding mutant strains.^41^, ^42^ Therefore, utilizing SP depended on the sequence from the most predominant circulating strain, such as Omicron, to generate a more efficient neutralization capacity could be a feasible approach. Strikingly, the antigen from Omicron BA.1 variant show reduced antigenicity,^43^, ^44^ and infection with Omicron could only induce limited cross‐neutralizing neutralizing activities.^12^ Nevertheless, another optimal solution for COVID‐19 vaccine design is selecting an antigen that can elicit a sustained immune response with broad‐spectrum cross‐reactivity. Previous studies suggested that the Beta SP might serve as a suitable antigen for vaccine development to elicit comprehensive immune responses.^14^, ^15^, ^45^ Individuals with a history of infection with the Beta variant exhibit vigorous cross‐neutralizing reactivity across SARS‐CoV‐2 VOCs.^46^ The mutations of K471N, E484K, and N501Y in the Beta spike may provide new epitopes, contributing to the cross‐neutralizing immunogenicity.^14^, ^16^ Other substitutions in Beta spike antigen might be imperative for the expanded breadth of immune response by well exhibiting the open or “up” configuration that exposes the RBD Ab epitopes.^14^, ^17^ Nevertheless, Omicron and its subvariants significantly escape from the neutralizing capacities induced by Beta variant. Besides the antigen of Beta variant, the subunit from Delta variant may be an alternative antigen for the design of the next‐generation COVID‐19 vaccine. Delta variant infection or Delta‐matched vaccine exhibit vaccination shows efficient cross‐reactivity neutralization.^12^, ^47^ We also previously reported a self‐assembly trimeric RBD protein vaccine derived from the Delta variant generated great titers of broad‐spectrum neutralizing Abs toward Omicron‐included variants.^18^ In terms of mechanism, our study reveals that mutations occurring at position L452R and T478K within RBD of the Delta variant confer advantages to the Ab response induced by vaccination against the Omicron. The T478K mutation, present in both Delta and Omicron, is essential for retaining protection toward the Omicron variant. Although the Omicron variants do not have the L452R mutation, our study shows that both the L452R and the Q493R mutations, which are specific to Delta and Omicron, are located on a compact two‐stranded β‐sheet within the exterior subdomain of the RBD. The presence of two amino acid substitutions leads to shared epitope characteristics that specifically target the β‐sheet. This facilitates the cross‐neutralization of antigens carrying the with Delta‐specific L452R mutation against Omicron.^18^ Echoing these observations, we thus utilized an adenovirus vector platform to engineer a chimeric vaccine by adding the RBD derived from Delta variant into the entire SP from Beta. As expected, both IM and IN immunization with Ad5‐Beta/Delta vaccine can induce sustained immune responses with cross‐reactivity across Omicron BA.5‐included SARS‐CoV‐2 VOCs. Thus, we believe that the Ad5‐Beta/Delta vaccine represents an optimal vaccine with high immunogenicity for eliciting broad cross‐neutralizing patterns to protect against not only the contemporary circulating variants but also past or future strains.

The currently approved COVID‐19 vaccines that predominantly produce considerable levels of sera IgG and strong systemic cellular immunity are almost administered via IM injection.^48^ Regarding the fact that the vaccines that are presently marketed effectively protect against symptomatic and severe infection, the capacity to curtail SARS‐CoV‐2 transmission is rather limited due to the poor mucosal immunity induced by the IM delivery of vaccine.^24^ It is believed that the respiratory mucosal IgA and T_RM_ cells play a crucial role as the first responders in safeguarding from SARS‐CoV‐2 infection throughout viral entry.^32^ Multiple investigations have manifested that administering adenovirus‐vector dependent COVID‐19 vaccines IN may stimulate strong mucosal immune responses that effectively guard toward SARS‐CoV‐2 infection and replication in the upper and lower respiratory tract.^26^, ^27^, ^28^, ^29^, ^30^, ^31^ However, in keeping with previous findings,^26^, ^29^, ^35^ our results, as shown in Figure 3, also indicated that IN immunization elicits a relatively lower systemic and cellular immune response than IM administration. Nevertheless, a combination of the IM and IN vaccination routes seems to represent an available approach to meet this challenge.^49^ Recent studies have revealed that initial IM vaccination accompanied by an IN booster resulted in the greatest levels of IgG, IgA, and neutralizing Abs.^50^ It also reported that utilizing integration of mucosal and systemic immunizations may elevate the persistence of CD8^+^T_RM_ cells in the lungs.^51^ In this research, we also showed that immunization with Ad5‐Beta/Delta vaccine via combining IM and IN routes conferred systemic and mucosal protective immunity versus Omicron‐included SARS‐CoV‐2 variant.

mRNA vaccines have received globe approval for emergency usage and have proven to be a platform for protection with high efficacy against symptomatic and severe infection.^19^, ^39^, ^48^ The mRNA‐1273 and BNT162B2 vaccines, which use the original form of the SP, provide substantial defense against severe cases of COVID‐19, hospitalization and death. The neutralization capacities generated in vaccinated individuals against Omicron‐included VOCs, are significantly dramatically compromised.^9^, ^52^, ^53^ Nevertheless, such a challenge should be solved through heterologous vaccination with a vaccine booster that is capable to induce enhanced broad cross‐reactivity immune response. In the current study, heterologous vaccination with Ad5‐Beta/Delta via IM or IN delivery both evoked improved humoral immune responses than homologous mRNA vaccination.^54^ It has been emerged that the integration of systemic mRNA vaccination in addition to a mucosal adenovirus‐based vaccine could elicit a stronger immune response than mRNA vaccination alone. In line with their results, the mucosal immunity, manifested by high levels of IgA and increased frequency of T_RM_ cells, could be generated by IN immunization with the chimeric vaccine followed by mRNA injection. Besides as the boost shot after mRNA vaccine, we also showed Ad5‐Beta/Delta vaccine could be an extra booster shot after subunit protein vaccine vaccination (Figure 4). Thus, the Ad5‐Beta/Delta vaccine could be used not only as an IN vaccine but also as a favorable extra heterologous booster shot to tackle the circulating Omicron and its subvariants by modifying the current immune response and enhancing its strength and scope.

In this investigation, we demonstrate that immunization with Ad5‐Beta/Delta provides effective protective immunity against pre‐Omicron VOCs and BA.5‐included Omicron subvariants. However, the investigation is constrained by the fact that we did not evaluate the neutralization induced by Ad5‐Beta/Deta vaccine toward recently emerged Omicron variants with remarkable immune escape, especially for XBB lineages. In addition, we solely utilized the pseudovirus and authentic neutralization assays to evaluate the broad‐spectrum neutralizing capacities against SARS‐CoV‐2 variants; however, detecting the viral loads in multiple tissues of the respiratory tract from animal models infected with live viruses after immunization may provide a more accurate evaluation of protective efficacy of the vaccine. Nevertheless, our study provides a novel strategy for creating an advanced COVID‐19 vaccine by using a chimeric antigen design to address the COVID‐19 epidemic.

## 4 MATERIALS AND METHODS

### 4.1 Materials

#### 4.1.1 Cell lines

The HEK293T cell cultures (ATCC CRL‐11268) and HEK293T cell cultures with the human angiotensin‐converting enzyme 2 (ACE2) receptor (ACE2/293T) cells^18^ were cultivated in Dulbecco's modified Eagle medium (DMEM) (Gibco) enriched with 10% fetal bovine serum (FBS), penicillin (100 U/mL), and streptomycin (100 mg/mL), and kept at 37°C with 5% CO_2_. HEK293 cells (ATCC CRL‐1573) were cultivated in DMEM medium supplemented with 10% FBS. Mycoplasma contamination was routinely monitored using the Mycoalert Mycoplasma Detection Kit (Lonza).

#### 4.1.2 Animals

Six to eight‐week‐old female BALB/c mice, which were specific pathogen free (SPF), were procured from Beijing Vital River Laboratory Animal Technologies Co., Ltd (Beijing, China). Seven‐week‐old female Sprague–Dawley (SD) rats were purchased from Beijing HFK Bioscience Co., Ltd. (China). A designated facility for SPF housing was established at the State Key Laboratory of Biotherapy, Sichuan University (Chengdu, Sichuan, China). The environment was regulated with a temperature range of 21−25°C, humidity between 30 and 70%, and a 12‐h dark/light cycle.

### 4.2 Method details

#### 4.2.1 Vaccine construction

The chimeric SP of the Beta and Delta SARS‐CoV‐2 variants was created by incorporating amino acid changes from RBD of the Delta variant into the whole SP of the Beta variant. The full SP with proline stabilizing mutations^55^ of wild‐type SARS‐CoV‐2 (GenBank YP_009724390.1), Beta (B.1.351, https://covariants.org/variants/20H.Beta.V2), Delta (B.1.617.2, https://covariants.org/variants/21A.Delta), Omicron (BA.1) (https://covariants.org/variants/21K.Omicron) variant. The SARS‐CoV‐2 variant, together with the chimeric Beta and Delta variants of SARS‐CoV‐2, underwent genetic modifications to increase their expression in human cell lines (Genewiz). Then, these adenoviral vectors expressing SPs were packaged and rescued in HEK293 cells through an AdMax adenovirus system (Microbix). Briefly, these spike genes were cloned into the adenovirus shuttle plasmid pDC316 to generate the recombinant pDC316‐S by Gibson assembly, respectively. HEK293 cells were cotransfected with the recombinant shuttle plasmid pDC316‐S and an E1/E3‐deficient Adenovirus‐5 genomic backbone plasmid pBHGlox(delta)E1,3Cre. The replication‐incompetent adenoviral vector vaccines were rescued and then amplified in HEK293 cells. Afterward, they underwent purification employing cesium chloride density‐gradient ultracentrifugation. In each adenoviral vector, the quantity of viral particles was measured employing ultraviolet spectrophotometry at 260 nm, as described.^29^ HEK293T cells were infected with Ad5‐WT, Ad5‐Beta, Ad5‐Delta, Ad5‐Omicron, and Ad5‐Beta/Delta, respectively, and each SP expression was discovered via ELISA with a SARS‐CoV‐2 Spike Protein ELISA kit (ACROBiosystems; Cat#RAS‐A039). In addition, the chimeric SP expression was verified by employed WB deploying an anti‐Spike Ab (Sino Biological; Cat#40591‐T62) after infection with Ad5‐Beta/Delta.

#### 4.2.2 Vaccinations of animals

BALB/c mice were immunized with three dosages of 5 × 10^9^ VPs of Ad5‐WT, Ad5‐Beta, Ad5‐Delta, Ad5‐Omicron, Ad5‐Beta/Delta vaccine, or empty adenovirus vector (Ad5‐Empty) via IN inoculation or IM injection on day 0, 28, and 56. SD rats were IN immunized with three doses of 2 × 10^10^ VPs of Ad5‐Beta/Delta vaccine or Ad5‐Empty following the same immunization program.

To ascertain the immune response stimulated via the integration of IM and IN immunization, BALB/c mice were inoculated with IM with Ad5‐Beta/Delta vaccine on days 0 and 28, and then received a third‐dose of IN vaccination on day 56 to assess the immunological response (2×IM+1×IN). Besides, mice were IM injected with one dose and followed by two doses of Ad5‐Beta/Delta via IN delivery (1×IM+2×IN). As a control, the mice were immunized with three dosages of the Ad5‐Beta/Delta vaccine via IM (3×IM) or IN (3×IN) delivery.

To ascertain the effectiveness of the Ad5‐Beta/Delta vaccine as a heterologous third‐dose booster to improve the immune response, BALB/c mice received three dosages of 5 μg mRNA/50 μL of encapsulated liposome (LPX)/Spike‐mRNA vaccine (3×mRNA) via IM injection. Alternatively, mice were received vaccinations IM with two doses of spike‐mRNA vaccine on days 0 and 28, then given three dose of Ad5‐Beta/Delta vaccine via IM injection (2×mRNA+IM Ad5‐Beta/Delta) or IN (2×mRNA+IN Ad5‐Beta/Delta) delivery on day 56 since the first vaccination. In the assay of sequential immunization after protein vaccine vaccination, BALB/c mice were IM immunized with 10 μg adjuvanted‐RBD‐HR/trimer on days 0 and 28, and IN administrated with Ad5‐Beta/Delta vaccine on day 56 (2×RBD‐HR+IN Ad5‐Beta/Delta). The control group consisted of mice that were either IM immunized with three doses of RBD‐HR/trimer (3×RBD‐HR) or IN vaccinated with three doses of Ad5‐Beta/Delta (3×IN Ad5‐Beta/Delta).

Sera from vaccinated animals were collected 3, 7, and 11 weeks after the first immunization to assay the binding and neutralizing Abs. The mice were slaughtered on day 14 following the third booster immunization to gather BALF, spleen, and lung tissues for assessing the cellular immune response and mucosal immunity induced by the Ad5‐Beta/Delta vaccine.

#### 4.2.3 Enzyme‐linked immunosorbent assay

Anti‐SARS‐CoV‐2 RBD‐specific IgG and IgA were identified by means of ELISA. To summarize, recombinant RBD proteins were applied to 96‐well plates (NUNC‐MaxiSorp; Thermo Fisher Scientific) containing a 1 μg/mL solution in carbonate‐bicarbonate buffer and incubated at 4°C overnight. Following that, the wells underwent three washes with a solution of 1×PBS with 0.1% Tween‐20 (PBST). This was subsequently followed by a 1‐h blocking period with PBST containing 1% BSA at ambient temperature. Serum or tracheal solutions that had been serially diluted in dilution buffer (100 μL/well) were introduced. After being incubated at a temperature of 37°C for 1 h, the wells underwent three rounds of washing with PBST. Subsequently, 100 μL of diluted horseradish peroxidase‐conjugated Abs were introduced to the wells. The Abs used were as follows: rabbit anti‐rat IgG (Abcam; Cat#ab6734), goat anti‐mouse IgG (Thermo Fisher Scientific; Cat#31430), goat anti‐mouse IgA (Abcam; Cat#ab97235), or goat anti‐rat IgA (Abcam; Cat#ab97185). The wells were rinsed three times after a 1‐h incubation at 37°C; they were subsequently developed employing 3,3′,5,5′‐tetramethyl biphenyl diamine for a duration of ten minutes at ambient temperature. Stopping the reaction with 100 μL/well of 1 M H_2_SO_4_ was achieved. In conclusion, the absorbance was quantified at 450 nm utilizing a microplate reader (Spectramax ABS; Molecular Devices). The highest possible dilution of serum that had an absorbance at least 2.1 times greater than the absorbance of the negative control serum was utilized to determine the endpoint titer.

#### 4.2.4 SARS‐CoV‐2 pseudovirus neutralization assay

A SARS‐CoV‐2 pseudovirus neutralization experiment was conducted on serum and BALF samples from vaccinated mice to ascertain their neutralizing activity.^18^ All SARS‐CoV‐2 pseudoviruses (GFP‐Luciferase) deployed in this investigation were obtained from Genomeditech company (China), and the titer of each pseudovirus has been detected by the company.

In brief, inactivated serum and BALF samples (60°C for 30 min) were threefold diluted, ranging from 6 to 65,610, followed by adding diluted pseudoviruses (50 μL/well) and coincubated at 37°C for 1 h. Afterward, a total of 12,000 293T/ACE2 cells were introduced into each well and kept at a temperature of 37°C for a period of 48 h in order to stimulate the production of luciferase. The supernatants were finally extracted, then a lysis reagent with luciferase substrate (100 μL/well) was added (Beyotime; Cat#RG005), and the amount of luminescence in 293T/ACE2 cells was measured using a multimode microplate reader (PerkinElmer, USA).

The positive control group was composed solely of viruses and cells, whereas the negative control group was consisted exclusively of cells. On the contrary, the sample group comprised not only viruses but also cells, as well as serum or nasal biopsy specimens. To calculate the proportion of neutralization, the following formula was applied:

$$\begin{array}{ccc} & & \mathrm{Neutralization}\;\left(\%\right)=\\ & & \;\left(\frac{\mathrm{luminescenc}e_{\mathrm{postive}}-\mathrm{luminescenc}e_{\mathrm{sample}}}{\mathrm{luminescenc}e_{\mathrm{postive}}-\mathrm{luminescenc}e_{\mathrm{negative}}}\right)\times 100\% \end{array}$$

#### 4.2.5 Live SARS‐CoV‐2 virus neutralization assay

We tested authentic viral neutralization tests using accepted procedures to assess the potential of sera to neutralize a wide variety of live SARS‐CoV‐2 viruses. The serum specimens were diluted and mixed with live SARS‐CoV‐2 viruses at a 50% concentration of tissue‐culture infectious doses (TCID50). Following the incubation for 1 h at 37°C, the mixture was introduced into 96‐well microplates containing Vero E6 cells at a concentration of 5 × 10^4^ cells per well. The cells were then incubated for a period of 72 h. Subsequently, the presence of cytopathogenic effects (CPE) was observed by microscopic examination. The levels of neutralizing Abs in the vaccinated serum were measured to estimate the 50% neutralization concentration (EC50).

#### 4.2.6 Antigenic cartography

An antigenic map was created using the antigenic cartography methodology, using the neutralization data of mouse sera and following established techniques.^36^, ^37^, ^38^ The construction of the antigenic map involved the utilization of the Racmacs package (version 1.2.4) in R. One thousand optimization steps were performed, a dilution step size of zero was employed, and the value ‘none’ was designated to the minimal column basis parameter. The distance between an antiserum point and an antigen point on the antigenic map is calculated by subtracting the concentration of antiserum toward the specific antigen from the log2 of the highest measured concentration of antiserum toward any antigen.

#### 4.2.7 Flow cytometry

The cells in BALF were collected to analyze the amount of T_RM_ cells in the trachea and lung tissue. Lymphocytes in BALF were stained for 30 min at 4°C with the following Abs: PerCP/Cyanine5.5‐conjugated anti‐mouse CD3 (BioLegend; Cat#100218), Brilliant Violet 421‐conjugated anti‐mouse CD4 (BioLegend; Cat#100438), Brilliant Violet 510‐conjugated anti‐mouse CD8 (BioLegend; Cat#100751), PE‐conjugated anti‐mouse CD44 (BioLegend; Cat#103008), FITC‐conjugated anti‐mouse CD69 (BioLegend; Cat#104506), and APC‐conjugated anti‐mouse CD103 Abs (BioLegend; Cat#121414). For assay of the formation of GCs in lymph nodes, Tfh and GC B cells were incubated with PerCP/Cyanine5.5‐conjugated anti‐mouse CD3, PE‐Cy7‐conjugated anti‐mouse CD45R/B220 (BioLegend; Cat#103208), Brilliant Violet 421‐conjugated anti‐mouse GL‐7 (BioLegend; Cat#144614), APC‐conjugated anti‐mouse CD95 (BioLegend; Cat#152604), Brilliant Violet 421‐conjugated anti‐mouse CD19 (BioLegend; Cat#115538), APC‐conjugated anti‐mouse CD4 (BioLegend; Cat#100412), PE‐conjugated anti‐mouse CXCR5 (BioLegend; Cat#145504), and Brilliant Violet 510‐conjugated PD‐1 Abs (BioLegend; Cat#135241).

Lung tissues were obtained from mice and cut into pieces smaller than 1 mm^3^ for intracellular cytokine labeling (ICS). The minced tissues were then digested in 6−8 mL of a digestion buffer containing collagenase I (1 mg/mL; Gibco, USA), collagenase IV (0.5 mg/mL; Gibco), and DNase I (40 U/mL; KeyGen Biotech) in DMEM medium (Gibco). After being incubated at 37°C for 1 h, single‐cell suspensions were generated by filtration via a 70 μm nylon mesh filter from Corning, USA, and then undergoing red blood cell lysis. Lymphocytes from the spleen have been extracted under sterile conditions, as mentioned before.^18^ Cultured lung mononuclear cells and splenic lymphocytes in 1640 complete medium containing 10% FBS, 1 mM pyruvate, 100 μg/mL streptomycin, 100 U/mL penicillin (all from Gibco), 20 U/mL IL‐2, and 50 μM β‐mercaptoethanol (all from Sigma–Aldrich). The cells were exposed to a concentration of 1 μg/mL of overlapping 15‐amino‐acid peptides that cover the SP. This was promptly followed by a 6‐h treatment with brefeldin A (Invitrogen; Cat#00‐4506‐51) to prevent the release of cytokines inside the cells. The cells were gathered and subjected to a variety of Abs incubation: PerCP/Cyanine5.5‐conjugated anti‐mouse CD3, APC‐conjugated anti‐mouse CD4, FITC‐conjugated anti‐mouse CD8 (BioLegend; Cat#100706), PE‐conjugated anti‐mouse CD44. Then, cells were fixed and permeabilized by Fixation/Permeabilization Kit with BD GolgiStop (BD Biosciences; Cat#554715), and incubated with PE‐Cy7‐conjugated anti‐mouse IFN‐γ (BioLegend; Cat#505826), Brilliant Violet 510‐conjugated anti‐mouse TNF‐α (BioLegend; Cat#506339) Abs for a duration of 1 h at ambient temperature. Figure S3 includes the gating techniques used in flow cytometry. The NovoCyte Flow Cytometer (ACEA Biosciences) was used to identify and analyze cells with the assistance of NovoExpress 1.4.1 software.

### 4.3 Statistics

The geometric mean values ± SD or mean values ± standard error of variation were used to represent the data, as specified in the figure captions. The statistical analyses were performed utilizing the GraphPad Prism 8.0 program. For comparisons between two groups, *p* Values were calculated using unpaired Student's *t*‐tests. For comparisons between multiple groups, one‐way or two‐way ANOVAs were performed, followed by Tukey's multiple comparison post hoc test. Significant *p* values were indicated by the notation ****p < 0.0001, ***p < 0.001, **p < 0.01, *p < 0.05, or ns (nonsignificant).

## AUTHOR CONTRIBUTIONS

X. W. originated and supervised the investigation and formulated the experimental designs. P. C. performed gene cloning, expression, and construction of adenovirus vaccines. X. S. prepared the mRNA vaccine. G. L. and Jiong Li prepared the MF59‐adjuvanted RBD‐HR/trimer vaccine. W. H., H. L., D. P., C. H., Yuhe Huang, and Jingyun Yang conducted vaccine formulation administered immunizations to animals and performed binding Ab assay and pseudovirus neutralization experiment. W. H., H. L., D. P., Yuhe Huang, C. H., Jian Liu, H. Q., A. A., Li Chen, J. A., F. Q., B. W., D. A., Z. Z., Ying Hao, Yu Zhang, Xiya Huang, C. Y., M. F., and Z. B. collected the serum and BALF samples, and performed flow cytometry to assay the frequencies of T_RM_, GCB and Tfh cells, and to evaluate the T cellular immune responses in lung and spleen tissues. Q. S., S. L., Yanan Zhou, and Youchun Wang performed a live SARS‐CoV‐2 neutralization assay. X. P. construed the antigenic map according to the neutralization results. X. W., W. H., H. L., D. P., Yuhe Huang, C. H., Jingyun Yang, Xuemei He, Xuejiao Han, M. L., H. H., W. C., H. D., Jian Lei, Lu Chen, X. Z., W. W., G. L., G. S., L. Y., Jinliang Yang, Jiong Li, and Z.W. involved in the data analysis and interpretation, in addition to providing assistance in adjusting the directions and interpreting the mechanistic aspects of the results. X. W., P. C., and W. H. wrote the manuscript. All authors have read and approved the final manuscript.

## CONFLICT OF INTEREST STATEMENT

The authors affirm that they do not have any financial interests or potential conflicts of interest to declare.

## ETHICS STATEMENT

This work was approved by Sichuan University's Institutional Animal Care and Use Committee for all animal studies, approval number (20220531057).

## Supporting information

Supporting Information

## Data Availability

All data that support the findings of this study are available in the main text and supplement information. All other relevant data are available from the lead contact upon reasonable request.

## References

[1] Cao Y , Yisimayi A , Jian F , et al. BA.2.12.1, BA.4 and BA.5 escape antibodies elicited by Omicron infection. Nature. 2022;608(7923):593‐602. 35714668 10.1038/s41586-022-04980-y PMC9385493

[2] Uriu K , Ito J , Zahradnik J , et al. Enhanced transmissibility, infectivity, and immune resistance of the SARS‐CoV‐2 omicron XBB.1.5 variant. Lancet Infect Dis. 2023;23(3):280‐281. 36736338 10.1016/S1473-3099(23)00051-8 PMC9889095

[3] Faraone JN , Qu P , Goodarzi N , et al. Immune evasion and membrane fusion of SARS‐CoV‐2 XBB subvariants EG.5.1 and XBB.2.3. Emerg Microbes Infect. 2023;12(2):2270069. 37819267 10.1080/22221751.2023.2270069 PMC10606793

[4] Kaku Y , Kosugi Y , Uriu K , et al. Antiviral efficacy of the SARS‐CoV‐2 XBB breakthrough infection sera against omicron subvariants including EG.5. Lancet Infect Dis. 2023;23(10):e395‐e396. 37708910 10.1016/S1473-3099(23)00553-4

[5] Ciccozzi A , Fiori PL , Casu M , Sanna D , Ciccozzi M , Scarpa F . The mutation point of view of the SARS‐CoV‐2 HV.1 lineage. J Med Virol. 2024;96(1):e29359. 38164631 10.1002/jmv.29359

[6] Liu L , Iketani S , Guo Y , et al. Striking antibody evasion manifested by the Omicron variant of SARS‐CoV‐2. Nature. 2022;602(7898):676‐681. 35016198 10.1038/s41586-021-04388-0

[7] Cao Y , Wang J , Jian F , et al. Omicron escapes the majority of existing SARS‐CoV‐2 neutralizing antibodies. Nature. 2022;602(7898):657‐663. 35016194 10.1038/s41586-021-04385-3 PMC8866119

[8] Planas D , Saunders N , Maes P , et al. Considerable escape of SARS‐CoV‐2 Omicron to antibody neutralization. Nature. 2022;602(7898):671‐675. 35016199 10.1038/s41586-021-04389-z

[9] Cele S , Jackson L , Khoury DS , et al. Omicron extensively but incompletely escapes Pfizer BNT162b2 neutralization. Nature. 2022;602(7898):654‐656. 35016196 10.1038/s41586-021-04387-1 PMC8866126

[10] Wang Q , Iketani S , Li Z , et al. Alarming antibody evasion properties of rising SARS‐CoV‐2 BQ and XBB subvariants. Cell. 2023;186(2):279‐286. e8. 36580913 10.1016/j.cell.2022.12.018 PMC9747694

[11] Miller J , Hachmann NP , Collier AY , et al. Substantial neutralization escape by SARS‐CoV‐2 omicron variants BQ.1.1 and XBB.1. N Engl J Med. 2023;388(7):662‐664. 36652339 10.1056/NEJMc2214314 PMC9878581

[12] Suryawanshi RK , Chen IP , Ma T , et al. Limited cross‐variant immunity from SARS‐CoV‐2 Omicron without vaccination. Nature. 2022;607(7918):351‐355. 35584773 10.1038/s41586-022-04865-0 PMC9279157

[13] Gagne M , Moliva JI , Foulds KE , et al. mRNA‐1273 or mRNA‐Omicron boost in vaccinated macaques elicits similar B cell expansion, neutralizing responses, and protection from Omicron. Cell. 2022;185(9):1556‐1571. e18. 35447072 10.1016/j.cell.2022.03.038 PMC8947944

[14] Sridhar S , Chicz RM , Warren W , et al. The potential of Beta variant containing COVID booster vaccines for chasing Omicron in 2022. Nat Commun. 2022;13(1):5794. 36184631 10.1038/s41467-022-33549-6 PMC9526810

[15] Lam JH , Shivhare D , Chia TW , et al. Artificial cell membrane polymersome‐based intranasal beta spike formulation as a second generation covid‐19 vaccine. ACS Nano. 2022;16(10):16757‐16775. 36223228 10.1021/acsnano.2c06350

[16] Wilks SH , Mühlemann B , Shen X , et al. Mapping SARS‐CoV‐2 antigenic relationships and serological responses. Science. 2023;382(6666):eadj0070. 37797027 10.1126/science.adj0070 PMC12145880

[17] Gobeil SM , Henderson R , Stalls V , et al. Structural diversity of the SARS‐CoV‐2 Omicron spike. Mol Cell. 2022;82(11):2050‐2068. e6. 35447081 10.1016/j.molcel.2022.03.028 PMC8947964

[18] He C , Yang J , Hong W , et al. A self‐assembled trimeric protein vaccine induces protective immunity against Omicron variant. Nat Commun. 2022;13(1):5459. 36115859 10.1038/s41467-022-33209-9 PMC9482656

[19] Baden LR , El Sahly HM , Essink B , et al. Efficacy and safety of the mRNA‐1273 SARS‐CoV‐2 vaccine. N Engl J Med. 2021;384(5):403‐416. 33378609 10.1056/NEJMoa2035389 PMC7787219

[20] Polack FP , Thomas SJ , Kitchin N , et al. Safety and efficacy of the BNT162b2 mRNA Covid‐19 vaccine. N Engl J Med. 2020;383(27):2603‐2615. 33301246 10.1056/NEJMoa2034577 PMC7745181

[21] Voysey M , Clemens SAC , Madhi SA , et al. Safety and efficacy of the ChAdOx1 nCoV‐19 vaccine (AZD1222) against SARS‐CoV‐2: an interim analysis of four randomised controlled trials in Brazil, South Africa, and the UK. Lancet (London, England). 2021;397(10269):99‐111. 33306989 10.1016/S0140-6736(20)32661-1 PMC7723445

[22] Logunov DY , Dolzhikova IV , Shcheblyakov DV , et al. Safety and efficacy of an rAd26 and rAd5 vector‐based heterologous prime‐boost COVID‐19 vaccine: an interim analysis of a randomised controlled phase 3 trial in Russia. Lancet (London, England). 2021;397(10275):671‐681. 33545094 10.1016/S0140-6736(21)00234-8 PMC7852454

[23] Sadoff J , Le Gars M , Shukarev G , et al. Interim results of a phase 1–2a trial of Ad26.COV2.S Covid‐19 vaccine. N Engl J Med. 2021;384(19):1824‐1835. 33440088 10.1056/NEJMoa2034201 PMC7821985

[24] Bleier BS , Ramanathan M Jr , Lane AP . COVID‐19 vaccines may not prevent nasal SARS‐CoV‐2 infection and asymptomatic transmission. Otolaryngol Head Neck Surg. 2021;164(2):305‐307. 33320052 10.1177/0194599820982633

[25] V'Kovski P , Kratzel A , Steiner S , Stalder H , Thiel V . Coronavirus biology and replication: implications for SARS‐CoV‐2. Nat Rev Microbiol. 2021;19(3):155‐170. 33116300 10.1038/s41579-020-00468-6 PMC7592455

[26] Afkhami S , D'Agostino MR , Zhang A , et al. Respiratory mucosal delivery of next‐generation COVID‐19 vaccine provides robust protection against both ancestral and variant strains of SARS‐CoV‐2. Cell. 2022;185(5):896‐915. e19. 35180381 10.1016/j.cell.2022.02.005 PMC8825346

[27] Hassan AO , Shrihari S , Gorman MJ , et al. An intranasal vaccine durably protects against SARS‐CoV‐2 variants in mice. Cell Rep. 2021;36(4):109452. 34289385 10.1016/j.celrep.2021.109452 PMC8270739

[28] Wu S , Zhong G , Zhang J , et al. A single dose of an adenovirus‐vectored vaccine provides protection against SARS‐CoV‐2 challenge. Nat Commun. 2020;11(1):4081. 32796842 10.1038/s41467-020-17972-1 PMC7427994

[29] Hassan AO , Kafai NM , Dmitriev IP , et al. A single‐dose intranasal ChAd vaccine protects upper and lower respiratory tracts against SARS‐CoV‐2. Cell. 2020;183(1):169‐184. e13. 32931734 10.1016/j.cell.2020.08.026 PMC7437481

[30] Cokarić Brdovčak M , Materljan J , Šustić M , et al. ChAdOx1‐S adenoviral vector vaccine applied intranasally elicits superior mucosal immunity compared to the intramuscular route of vaccination. Eur J Immunol. 2022;52(6):936‐945. 35304741 10.1002/eji.202249823 PMC9087383

[31] Cao H , Mai J , Zhou Z , et al. Intranasal HD‐Ad vaccine protects the upper and lower respiratory tracts of hACE2 mice against SARS‐CoV‐2. Cell Biosci. 2021;11(1):202. 34879865 10.1186/s13578-021-00723-0 PMC8653804

[32] Lapuente D , Fuchs J , Willar J , et al. Protective mucosal immunity against SARS‐CoV‐2 after heterologous systemic prime‐mucosal boost immunization. Nat Commun. 2021;12(1):6871. 34836955 10.1038/s41467-021-27063-4 PMC8626513

[33] Wang Z , Lorenzi JCC , Muecksch F , et al. Enhanced SARS‐CoV‐2 neutralization by dimeric IgA. Sci Transl Med. 2021;13(577):eabf1555. 33288661 10.1126/scitranslmed.abf1555 PMC7857415

[34] Rakhra K , Abraham W , Wang C , et al. Exploiting albumin as a mucosal vaccine chaperone for robust generation of lung‐resident memory T cells. Sci Immunol. 2021;6(57):eabd8003. 33741657 10.1126/sciimmunol.abd8003 PMC8279396

[35] Feng L , Wang Q , Shan C , et al. An adenovirus‐vectored COVID‐19 vaccine confers protection from SARS‐COV‐2 challenge in rhesus macaques. Nat Commun. 2020;11(1):4207. 32826924 10.1038/s41467-020-18077-5 PMC7442803

[36] Mykytyn AZ , Rissmann M , Kok A , et al. Antigenic cartography of SARS‐CoV‐2 reveals that Omicron BA.1 and BA.2 are antigenically distinct. Sci Immunol. 2022;7(75):eabq4450. 35737747 10.1126/sciimmunol.abq4450 PMC9273038

[37] Smith DJ , Lapedes AS , de Jong JC , et al. Mapping the antigenic and genetic evolution of influenza virus. Science. 2004;305(5682):371‐376. 15218094 10.1126/science.1097211

[38] Mykytyn AZ , Rosu ME , Kok A , et al. Antigenic mapping of emerging SARS‐CoV‐2 omicron variants BM.1.1.1, BQ.1.1, and XBB.1. Lancet Microbe. 2023;4(5):e294‐e295. 36657480 10.1016/S2666-5247(22)00384-6 PMC9842387

[39] Haas EJ , Angulo FJ , McLaughlin JM , et al. Impact and effectiveness of mRNA BNT162b2 vaccine against SARS‐CoV‐2 infections and COVID‐19 cases, hospitalisations, and deaths following a nationwide vaccination campaign in Israel: an observational study using national surveillance data. Lancet (London, England). 2021;397(10287):1819‐1829. 33964222 10.1016/S0140-6736(21)00947-8 PMC8099315

[40] Shuai H , Chan JF , Hu B , et al. Attenuated replication and pathogenicity of SARS‐CoV‐2 B.1.1.529 Omicron. Nature. 2022;603(7902):693‐699. 35062016 10.1038/s41586-022-04442-5

[41] He C , Yang J , He X , et al. A bivalent recombinant vaccine targeting the S1 protein induces neutralizing antibodies against both SARS‐CoV‐2 variants and wild‐type of the virus. MedComm. 2021;2(3):430‐441. 34226895 10.1002/mco2.72 PMC8242662

[42] Laurie MT , Liu J , Sunshine S , et al. SARS‐CoV‐2 variant exposures elicit antibody responses with differential cross‐neutralization of established and emerging strains including Delta and Omicron. J Infect Dis. 2022;225(11):1909‐1914. 34979030 10.1093/infdis/jiab635 PMC8755395

[43] He C , He X , Yang J , et al. Spike protein of SARS‐CoV‐2 Omicron (B.1.1.529) variant have a reduced ability to induce the immune response. Signal Transduct Target Ther. 2022;7(1):119. 35397623 10.1038/s41392-022-00980-6 PMC8994023

[44] Tubiana J , Xiang Y , Fan L , et al. Reduced B cell antigenicity of Omicron lowers host serologic response. Cell Rep. 2022;41(3):111512. 36223774 10.1016/j.celrep.2022.111512 PMC9515332

[45] Launay O , Cachanado M , Luong Nguyen LB , et al. Immunogenicity and safety of beta‐adjuvanted recombinant booster vaccine. N Engl J Med. 2022;387(4):374‐376. 35767474 10.1056/NEJMc2206711 PMC9258749

[46] Moyo‐Gwete T , Madzivhandila M , Makhado Z , et al. Cross‐reactive neutralizing antibody responses elicited by SARS‐CoV‐2 501Y.V2 (B.1.351). N Engl J Med. 2021;384(22):2161‐2163. 33826816 10.1056/NEJMc2104192 PMC8063886

[47] Qu L , Yi Z , Shen Y , et al. Circular RNA vaccines against SARS‐CoV‐2 and emerging variants. Cell. 2022;185(10):1728‐1744. e16. 35460644 10.1016/j.cell.2022.03.044 PMC8971115

[48] Sadarangani M , Marchant A , Kollmann TR . Immunological mechanisms of vaccine‐induced protection against COVID‐19 in humans. Nat Rev Immunol. 2021;21(8):475‐484. 34211186 10.1038/s41577-021-00578-z PMC8246128

[49] Tiboni M , Casettari L , Illum L . Nasal vaccination against SARS‐CoV‐2: synergistic or alternative to intramuscular vaccines? Int J Pharm. 2021;603:120686. 33964339 10.1016/j.ijpharm.2021.120686 PMC8099545

[50] Li X , Wang L , Liu J , et al. Combining intramuscular and intranasal homologous prime‐boost with a chimpanzee adenovirus‐based COVID‐19 vaccine elicits potent humoral and cellular immune responses in mice. Emerg Microbes Infect. 2022;11(1):1890‐1899. 35775819 10.1080/22221751.2022.2097479 PMC9331206

[51] Lavelle EC , Ward RW . Mucosal vaccines—fortifying the frontiers. Nat Rev Immunol. 2022;22(4):236‐250. 34312520 10.1038/s41577-021-00583-2 PMC8312369

[52] Planas D , Saunders N , Maes P , et al. Considerable escape of SARS‐CoV‐2 Omicron to antibody neutralization. Nature. 2021. 10.1038/s41586-021-04389-z 35016199

[53] Nemet I , Kliker L , Lustig Y , et al. Third BNT162b2 vaccination neutralization of SARS‐CoV‐2 Omicron infection. N Engl J Med. 2021. 10.1056/NEJMc2119358 PMC8823651 34965337

[54] Tang J , Zeng C , Cox TM , et al. Respiratory mucosal immunity against SARS‐CoV‐2 after mRNA vaccination. Sci Immunol. 2022;7(76):eadd4853. 35857583 10.1126/sciimmunol.add4853 PMC9348751

[55] Wrapp D , Wang N , Corbett KS , et al. Cryo‐EM structure of the 2019‐nCoV spike in the prefusion conformation. Science. 2020;367(6483):1260‐1263. 32075877 10.1126/science.abb2507 PMC7164637

