# Supplementary material for: A chimeric adenovirus‐vectored vaccine based on Beta spike and Delta RBD confers a broad‐spectrum neutralization against Omicron‐included SARS‐CoV‐2 variants
Source: MedComm (2020). 2024 Apr 27;5(5):e539. doi: 10.1002/mco2.539 (PMC11055958; doi:10.1002/mco2.539)
Supplement: Supplementary file 1 — Supporting Information [file MCO2-5-e539-s001.pdf]

Supplementary Materials for

A chimeric adenovirus-vectored vaccine based on Beta spike and Delta RBD confers a broad protective immunity against Omicron-included SARS-CoV-2 variants

This PDF file includes:

Figure S1 to S3

Supplemental Figures 1-3

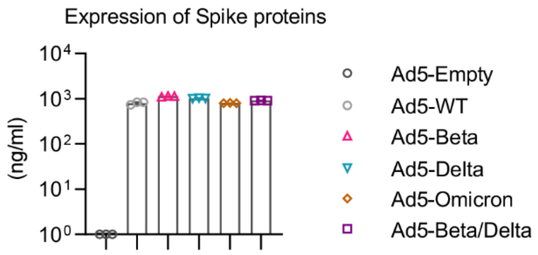

Figure S1. Expression of spike proteins after infection with adenovirus-vectored vaccines. HEK293T cells were infected with Ad5-Empty, Ad5-WT, Ad5-Beta, Ad5-Delta, Ad5-Omicron and Ad5-Beta/Deta, respectively. The expression of spike proteins 48 hours after infection were determined by ELISA assay. Data are presented as mean values ± SEM.

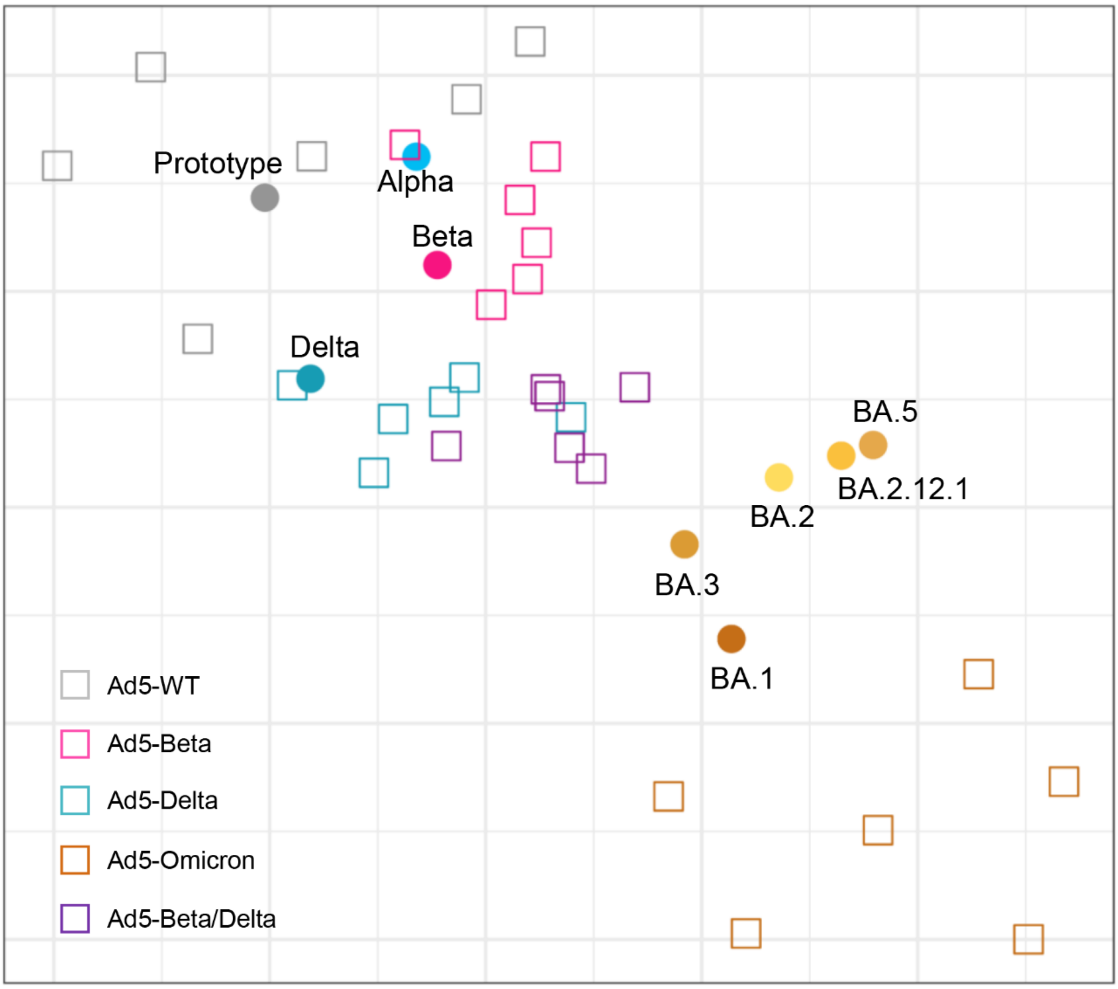

Figure S2. Antigenic map comparing neutralization against SARS-CoV-2 pseudoviruses. The antigenic map was constructed based on the sera neutralization data from Figure 1E.

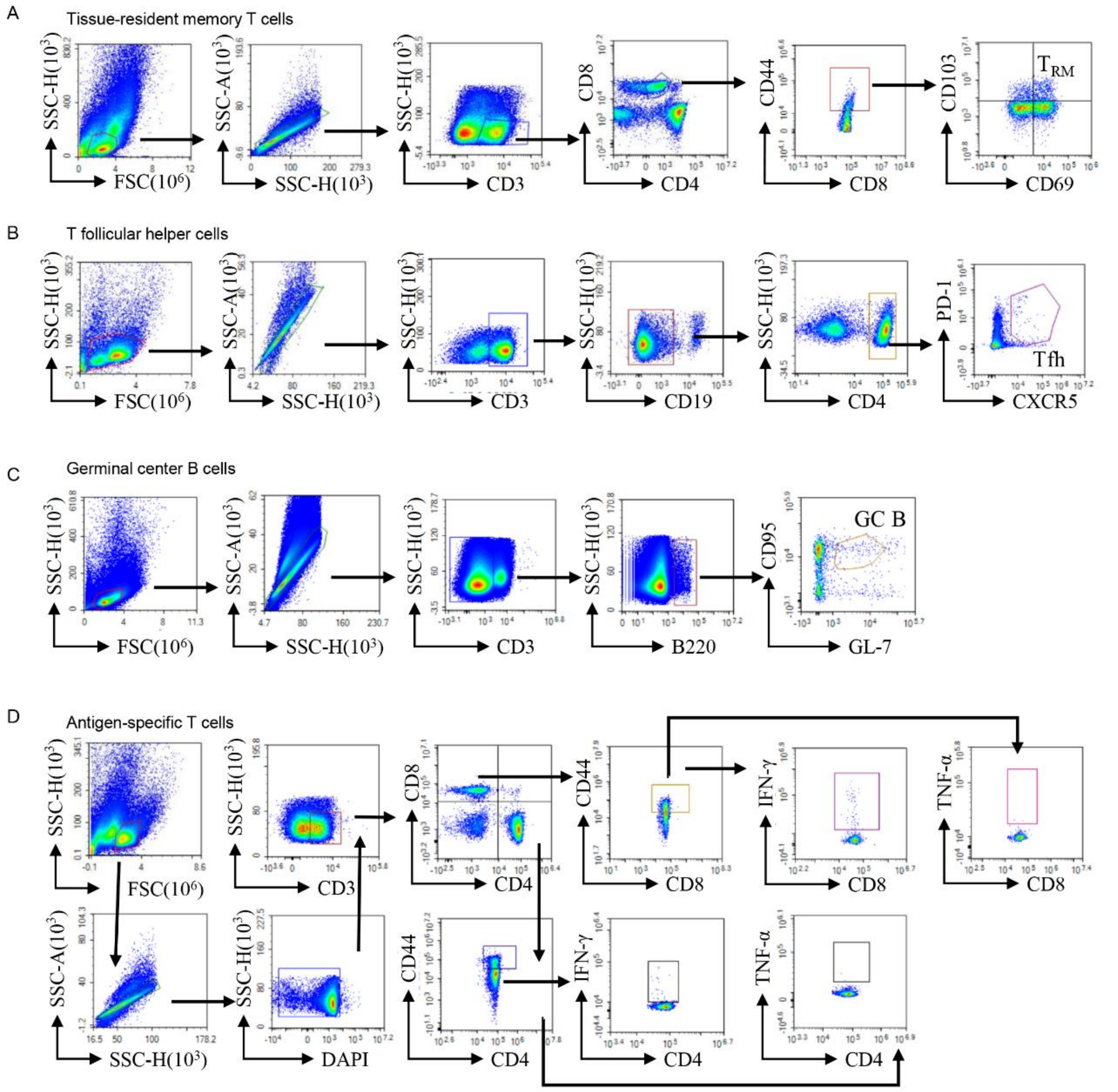

Figure S3. Gating strategy and flow cytometry analysis of immune cells in vivo.

(A) Tissue-resident memory T cells (T_{RM}) were identified as CD3^{+}CD8^{+}/CD4^{+}CD44^{+}CD69^{+}CD103^{+} cells.

(B) T follicular helper (Tfh) cells were identified as CD19^{-}CD3^{+}CD4^{+}CXCR5^{+}PD-1^{+} cells.

(C) Germinal center B (GC B) cells were identified as CD3^{-}B220^{+}GL-7^{+} cells.

(D) Antigen-specific T cells were identified as CD3^{+}CD8^{+}/CD4^{+}CD44^{+}IFN-γ^{+} or TNF-α^{+} cells after stimulation with peptide pools covering spike proteins.
